# Tiamulin-Resistant Mutants of the Thermophilic Bacterium *Thermus thermophilus*

**DOI:** 10.3390/antibiotics9060313

**Published:** 2020-06-09

**Authors:** Erin E. Killeavy, Gerwald Jogl, Steven T. Gregory

**Affiliations:** 1 Department of Cell and Molecular Biology, The University of Rhode Island, Kingston, RI 02881, USA; erin_killeavy@my.uri.edu; 2 Department of Molecular Biology, Cell Biology and Biochemistry, Brown University, Providence, RI 02912, USA; gerwald_jogl@brown.edu

**Keywords:** tiamulin, peptidyltransferase, ribosome, rRNA, ribosomal protein, antibiotic resistance mutation, *Thermus thermophilus*

## Abstract

Tiamulin is a semisynthetic pleuromutilin antibiotic that binds to the 50S ribosomal subunit A site and whose (((2-diethylamino)ethyl)thio)-acetic acid tail extends into the P site to interfere with peptide bond formation. We have isolated spontaneous tiamulin-resistant mutants of the thermophilic bacterium *Thermus thermophilus*, containing either single amino acid substitutions in ribosomal protein uL3 or single base substitutions in the peptidyltransferase active site of 23S rRNA. These mutations are consistent with those found in other organisms and are in close proximity to the crystallographically determined tiamulin binding site. We also conducted a cross-resistance analysis of nine other single-base substitutions in or near the peptidyltransferase active site, previously selected for resistance to structurally unrelated antibiotics. While some of the base substitutions in 23S rRNA are positioned to directly affect tiamulin-ribosome contacts, others are some distance from the tiamulin binding site, indicating an indirect mechanism of resistance. Similarly, amino acid substitutions in uL3 are predicted to act indirectly by destabilizing rRNA conformation in the active site. We interpret these observations in light of the available ribosome X-ray crystal structures. These results provide a more comprehensive profile of tiamulin resistance caused by mutations in the bacterial ribosome.

## 1. Introduction

The ribosomal peptidyltransferase active site (also referred to as the PTC), the site of peptide bond formation during protein synthesis, is the target of a number of structurally unrelated classes of antibiotics, and mutations conferring resistance to many of these drugs have been identified in the genes encoding 23S rRNA or ribosomal proteins from a wide range of organisms. Advances in structural biology have led to numerous high-resolution structures of ribosomes in complex with antibiotics, providing a wealth of information about the mechanisms of action of these important drugs and facilitating predictions about mechanisms of antibiotic resistance reviewed by [1,2,3].

The pleuromutilin class of antibiotics, the first of which was isolated from the fungus *Pleurotus mutilis* [4], includes the semi-synthetic derivatives tiamulin, repatamulin, and valnemulin, and are used primarily in veterinary medicine reviewed by [5]. Based on chemical footprinting of 23S rRNA in *Escherichia coli* ribosomes, pleuromutilins bind to the PTC and compete for binding with the 16-atom macrolide and peptidyltransferase inhibitor carbomycin (though not with the 14-atom macrolide erythromycin) [6], and thereby inhibit peptide bond formation [7]. The precise nature of pleuromutilin binding to the ribosome was subsequently determined by X-ray crystallography of ribosome–drug complexes [8,9,10]. Tiamulin interacts exclusively with rRNA residues, although ribosomal protein uL3 is in close proximity to the tiamulin binding site without making direct contact with the drug.

Tiamulin-resistant mutants with altered ribosomal proteins were first identified in *E. coli*, although the exact nature of the mutations was not determined [11]. Subsequent studies of several organisms, including *E. coli* [12,13], *Brachyspira* spp. [14], and *Staphylococcus aureus* [15,16], found resistance to result from amino acid substitutions in ribosomal protein uL3. Ribosomal protein uL3 consists of a globular domain situated on the 50S subunit surface, and an extended loop that reaches deep into the subunit where it approaches the PTC [17,18,19,20]. Amino acid substitutions conferring tiamulin resistance occur in this loop and presumably confer resistance by affecting local rRNA conformation. Resistance results from reduced binding, as indicated by chemical footprinting of mutant ribosomes from *Brachyspira* spp. [14]. Consistent with chemical probing data, tiamulin-resistance also results from base substitutions in 23S rRNA in and around the PTC of several organisms, including *E. coli* [12], *Brachyspira* spp. [14], *S*. *aureus* [16], *Mycobacterium smegmatis* [21], and *Mycoplasma gallisepticum* [22].

Both the chemical probing data and the nature of mutations can to some extent be rationalized given the X-ray crystal structures of pleuromutilins in complex with ribosomes from either the bacterium *Deinococcus radiodurans* [8,9] or the archaeon *Haloarcula marismortui* [10]. Several base substitutions in 23S rRNA occur at sites of drug contact and presumably lead to reduced binding affinity. Amino acid substitutions in uL3 occur at residues in close proximity to some of these same 23S rRNA residues, suggesting indirect effects on RNA conformation as the mechanism of resistance. While binding of pleuromutilins protects some bases (U2506, U2584, U2585) in close proximity to the drug from chemical probes, it enhances reactivity of others (A2058 and A2059), more distant from the drug binding site, suggesting that drug binding can induce conformational changes [6]. It also suggests that substitutions of bases not in the immediate vicinity of tiamulin could confer drug resistance by indirect means.

In order to more extensively characterize the basis for pleuromutilin resistance, we isolated spontaneous tiamulin-resistant mutants of the thermophilic bacterium *Thermus thermophilus* and tested for cross-resistance to tiamulin a number of mutants with base substitutions in the PTC previously selected for resistance to other antibiotics [23]. This organism has proven to be a good model system for the study of antibiotic-resistance mutations with the ability to combine facile genetics, biochemistry, and crystallography of its ribosomes [24]. Building upon previous studies, these data provide a more comprehensive examination of tiamulin-resistance. By examining published crystal structures of tiamulin bound to the 50S subunit of *D. radiodurans* [8] and *H. marismortui* [10], we assess the likely mechanism of resistance caused by these mutations. While base substitutions occur in the tiamulin binding pocket, others are located some distance away, supporting the hypothesis that pleuromutilin resistance can be caused by conformational changes due to mutations distant from the drug binding site, and that these mutations distort ribosome conformation.

## 2. Results

### 2.1. Isolation of Spontaneous Tiamulin-Resistant Mutants

In order to maximize the range of mutants arising in selections, we employed two different *T. thermophilus* strains, HB27 [25] and IB-21 [26], originally isolated from locations geographically remote from one another (Japan and Iceland, respectively) and having different optimal growth temperatures. These strains both contain two copies of each rRNA gene, but rapid gene conversion generally results in homoallelic mutants producing pure populations of mutant ribosomes [23]. We noted that temperature, strain, drug concentration, and media formulation, are all variables potentially influencing the spectrum of mutations isolated (our unpublished observations). We therefore selected tiamulin-resistant mutants at either 65 or 72 °C and at 50, 100, or 200 μg/mL. Mutants and their selections are listed in Table 1.

**Figure 1:**
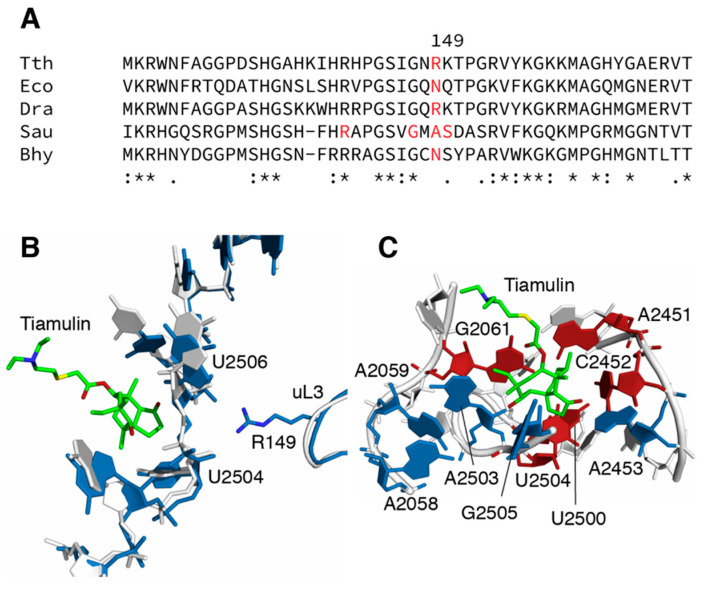
Sites of mutations and inferred mechanisms of resistance. (**A**) Sequence alignment of ribosomal protein uL3 encompassing the sites of mutations identified in this study. Uses organisms for whom tiamulin-resistance mutations in uL3 have been found. Tth, *Thermus thermophilus* (Accession number Q72I04); Dra, *Deinococcus radiodurans* (Accession number Q9RXK2); Eco, *Escherichia coli* (Accession number P60438); Sau, *Staphylococcus aureus* (Accession number P60449); Bhy, *Brachyspira hyodysenteriae* (Accession number A0A2K9JGD3) (**B**) Interaction of ribosomal protein uL3 and 23S rRNA at the tiamulin binding site, based on the crystal structures of ribosomes from *T. thermophilus* (PDB entry 4y4p.cif; 36) and *D. radiodurans* (PDB entry 1xbp.cif; 8). Structures were aligned and images rendered using PyMOL (Schrodinger). R149 is within hydrogen bonding distance of the 2’ hydroxyl of U2504 and the phosphate oxygen of U2506. (**C**) Interactions between tiamulin and 23S rRNA in the *D. radiodurans* (PDB entry 1xbp.cif; 8) 50S subunit structure. Sites of mutation conferring resistance are colored red, sites of mutations not conferring resistance are colored blue.

Based on the known binding site of tiamulin in the PTC, as well as previously described tiamulin-resistant mutants, we PCR amplified and sequenced the corresponding regions of the *rrlA* and *rrlB* genes encoding 23S rRNA and the *rplC* gene encoding ribosomal protein uL3. We identified two mutations in *rplC*, CGC to either CAC or TGC, that result in either of two amino acid substitutions at the same position, R149H or R149C of uL3 (*E. coli* numbering used throughout; corresponding to R144 in the *T. thermophilus* sequence; Figure 1a). These were selected on 50 and 100 μg/mL tiamulin. Amino acid substitutions at this and surrounding positions have been identified in other species [12,13,14,15,16]. We also identified a total of six different base substitutions in the 23S rRNA in the PTC. Among these were G2061A, G2061U, A2451U, C2452U, U2500A, and U2504G. We had previously identified several of these (G2061A, C2452U, U2500A, and U2504G) in selections for mutants resistant to chloramphenicol [23], but have not previously observed the G2061U and A2451U substitutions in *T. thermophilus*. A2451U is particularly noteworthy as having been described previously only in mouse mitochondria [27] and *Mycobacterium smegmatis* [28].

To demonstrate a direct causal relationship between mutation and resistance phenotype, wild-type *T. thermophilus* was transformed with genomic DNA from the resistant mutants, selecting tiamulin resistance and then confirming the presence of the original mutation by sequencing. In all cases, transformants contained the originally selected tiamulin-resistance mutation, indicating a causal relationship between mutation and phenotype.

### 2.2. Resistance Phenotypes

We estimated comparative resistance to tiamulin using a disc diffusion assay (Table 2; see Section 4). As controls, assays were also performed using the 30S subunit antibiotic streptomycin, the DNA gyrase inhibitor ciprofloxacin, and the cell wall synthesis inhibitor ampicillin. These analyses provide a crude, semi-quantitative measure of relative drug resistance. Ribosomal protein uL3 mutants conferred the lowest level of tiamulin resistance, exhibiting significant inhibition zones, while all of the 23S rRNA mutants were generally more resistant. For instance, G2061A, G2061U, A2451U, U2500, and U2504G showed no zones of inhibition. The possibility that lack of inhibition due to thermal degradation of tiamulin during prolonged incubation of slow-growing mutants can be excluded since discs pre-incubated at 72 °C for 1 week show no decrease in inhibition zone against wild-type *T. thermophilus*.

We examined the cross-resistance phenotypes of PTC mutants to various 50S subunit inhibitors binding at or near the peptidyltransferase active site. In addition to tiamulin, we tested sensitivity to the 14-atom macrolides erythromycin, clarithromycin, roxithromycin, the 15-atom macrolide azithromycin, the 16-atom macrolides midecamycin, tylosin, and spiramycin, the lincosamide lincomycin, and chloramphenicol. Ampicillin and the DNA gyrase inhibitor ciprofloxacin were included for comparison. Results from these tests are shown in Table 2.

The uL3 mutations produce no cross resistance to other drugs that bind to this region and were specific for tiamulin. We previously identified chloramphenicol-resistant or macrolide-resistant mutants of *T. thermophilus* containing single base substitutions in the vicinity of the PTC [23]. As several of these same base substitutions were identified in selections for tiamulin-resistant mutants in the current study, we examined a number of available PTC mutants for resistance to tiamulin. These included A2058G, A2059G, G2447A, A2453G, U2500C, A2503G, U2504C, U2504A, and G2505A. Of these, the only ones found to confer tiamulin resistance were G2447A and U2504A, which confer weak resistance (16 and 17 mm zones of inhibition, respectively), at levels comparable to those produced by the uL3 mutations. Since U2504 was identified in our initial selections, only G2447A was not identified in selections for tiamulin resistance. This suggests that our initial selections were close to exhaustive.

### 2.3. Resistance to Other PTC-Binding Antibiotics

A large number of antibiotics target the PTC, and we examined our tiamulin-resistant mutants for cross-resistance to a broad range of drugs. Most notable is the consistent hypersensitivity of the G2061A mutant to macrolides, but not to tiamulin, lincomycin, or chloramphenicol. While this could be accounted for as a result of the extremely slow growth rate of this mutant, the A2451U mutant does not show this same level of hypersensitivity, despite also being very slow growing. This latter mutant shows hypersensitivity to the 16-atom macrolide tylosin and resistance to lincomycin and chloramphenicol. The isolation of A2451U as a tiamulin-resistance mutation showing cross resistance to chloramphenicol is perhaps significant as base substitutions at A2451 have previously been isolated as chloramphenicol-resistance mutations in mouse mitochondria [27] and found to confer chloramphenicol resistance after site-directed mutagenesis of *Mycobacterium smegmatis* [28].

Base substitutions at U2504 produce varying phenotypes, depending on the actual base substitution. Thus, U2504G confers complete tiamulin resistance (and was identified as a spontaneous tiamulin-resistant mutant), while U2504A confers minimal resistance and U2504C confers no resistance at all. Base substitutions at U2504 also exhibit varied responses to either lincomycin or the 16-atom macrolide tylosin, with U2504G conferring resistance, U2504C conferring only weak resistance, and U2504A conferring hypersensitivity. In contrast, all three base substitutions confer chloramphenicol resistance and were previously identified in selections for chloramphenicol resistance [23].

## 3. Discussion

Amino acid substitutions in uL3 conferring tiamulin resistance have been found in several organisms, including *E. coli*, *S. aureus*, and *Brachyspira* spp. Currently there are crystal structures of tiamulin bound to ribosomes from two sources, *D. radiodurans* [8] and *H. marismortui* [10]. In neither case is uL3 positioned to make direct contact with tiamulin (Figure 1b), indicating that the mechanism of resistance conferred by uL3 mutations is indirect, presumably via some perturbation of rRNA conformation. While the *D. radiodurans* structure does not show sidechains for ribosomal protein uL3 [8], structures of the *T. thermophilus* ribosome shows the δ-guanidinium group of R149 of uL3 to be within 3 Å of a phosphate oxygen of U2506 of 23S rRNA [29]. A site-directed mutagenesis study of *E. coli* uL3 [13] found that, while N149S and N149D substitutions confer resistance (the latter confirming previous studies of spontaneous mutants), N149R does not, consistent with the presence of an arginine at this position in the wild-type *T. thermophilus* uL3. However, examination of the crystal structure of the *E. coli* ribosome [20] or the cryo-EM structure of the *S. aureus* ribosome [30] indicates that neither the asparagine residue at position 149 of *E. coli* nor the alanine at position 149 of *S. aureus* uL3 are in position to make a direct contact with 23S rRNA. Other amino acid substitutions found in uL3 of tiamulin-resistant *S. aureus* are R141S, G147R, A149L, S150L, and D151Y [15,16]. Therefore, the means by which mutations at N149 of *E. coli* or A149 of *S. aureus* uL3 confer resistance is not clear, as neither of these residues are in position to interact with 23S rRNA as does the arginine in *T. thermophilus* uL3.

All of the base substitutions conferring tiamulin resistance occur at universally or nearly universally conserved positions (Table 3), suggesting that each of these residues plays an important role in catalysis, rRNA folding, or stabilizing the structure in a catalytically active conformation. It therefore follows that base substitutions in the active site could perturb the structure sufficiently to impact tiamulin binding affinity. There is currently no crystal structure of the *T. thermophilus* ribosome in complex with tiamulin, so structural interpretations are based on examination of crystal structures of the *D. radiodurans* [8] and *H. marismortui* [10] 50S subunit–tiamulin complexes.

While the exact effects of base substitutions on the structure of rRNA in the PTC are difficult to predict, all the mutants are viable, indicating that the conformation of the mutant active sites must sufficiently resemble the native structure to permit catalysis of peptide bond formation. What the crystal structures do provide is an approximation of the closest approach of mutated residues and tiamulin (Table 3). While some differences between the two structures are evident, some interpretation regarding mechanisms of resistance is still possible.

There is a rough correlation between the proximity of mutated residues to tiamulin and the resistance phenotype; mutated residues near the tiamulin binding site are more likely to cause resistance than mutated residues at more remote positions (Figure 1c). Thus, substitutions at A2058 and A2059, which are between 6 and 7 Å from tiamulin, fail to confer resistance, leaving unresolved the question of enhanced chemical reactivity of A2058 upon tiamulin binding [6]. It is worth noting that U2504G confers resistance to both tiamulin and chloramphenicol, while disrupting the contact of uL3 with the nearby U2506 confers only tiamulin resistance, suggesting that the latter mutation has less of an impact on the local structure. This is also consistent with the higher level of tiamulin resistance conferred by the U2504G substitution.

A2451 is the 23S rRNA residue closest to the site of peptide bond formation, as first revealed by the crystal structure of the *H. marismortui* 50S subunit [31]. A2451 is a universally conserved nucleotide [32] and only two other instances of viable mutants having base substitutions at this position have been reported [27,28]; substitutions at A2451 produce dominantly lethal phenotypes in *Escherichia coli* [33]. A2451 is under 4 Å from tiamulin but does not appear to make direct contact. A2503G and G2505A, originally isolated as chloramphenicol-resistance mutations, do not confer tiamulin resistance despite being within 3 Å of tiamulin. The nearest approach to tiamulin by A2503 is via the C8, and would thus not be influenced by an A to G transition. The nearest approach from G2505 is via the backbone, and similar would not necessarily be affected by a base substitution.

Several of the mutations cannot be explained by direct loss of antibiotic–ribosome contact. G2447’s closest approach to tiamulin is about 5 Å and U2500 roughly 7 Å. Substitutions at these positions could perturb the structure sufficiently to diminish binding without propagating throughout the active site, and thereby having drastic effects on peptide-bond forming ability. The ability of base substitutions in the PTC to confer resistance despite being remote from the drug binding site has been observed before [34,35]. The idea that pleuromutilins bind via an induced-fit mechanism has been proposed based on X-ray crystal structures [35]. The enhanced reactivity of A2058 to chemical probes suggests an indirect influence of tiamulin on the conformation of these residues. These observations further emphasize the ability of antibiotic-resistance mutations to exert their effects from a position remote from the antibiotic binding site. More detailed structural analyses will be necessary to uncover the precise basis for resistance caused by individual mutations.

## 4. Materials and Methods

### 4.1. Bacterial Strains and Cultivation

All mutants were derived from the *T. thermophilus* strains HB27 (ATCC BAA-163) [25] or IB-21 [26]. All strains were grown in TEM (Thermus Enhanced Medium, ATCC Medium 1598) or plated on TMG medium (identical to TEM with the exception of 0.5 g/L CaCl_2_ and lacking phosphate buffer) solidified with Gellam Gum (PlantMedia). Antibiotics were from the author’s collection or were purchased from Sigma-Aldrich Chemical Corp.

### 4.2. Isolation of Spontaneous Mutants

To isolate mutants, overnight cultures originating from single colonies were grown in TEM overnight to saturation, and 10^9^ cells were plated onto TMG plates containing tiamulin at 50, 100, or 200 μg/mL. Mutants derived from either of the two *T. thermophilus* strains, IB-21 or HB27, were isolated at 62, 65, or 72 °C. After several days, mutant colonies appearing on selection plates were streaked for isolation on the same selection medium. Colonies arising on restreaks were streaked a second time on TMG plates without antibiotic. Single colonies were used to inoculate overnight cultures in TEM, grown to saturation, archived as glycerol stocks, and harvested for genomic DNA preps using the Wizard Genomic DNA kit (Promega) or the PureLink genomic DNA kit (Invitrogen).

### 4.3. Analysis of Mutants

PCR amplification of the *rplC* locus or the PTC region of *rrlA* and *rrlB* was performed using OneTaq DNA Polymerase (New England Biolabs). The *rplC* locus encoding ribosomal protein uL3 was amplified using oligonucleotide primers (IDT) Tth_rplC-fwd1 (5′-GAGCACTTTGAGCTGCGCACCCACAACC-3′) and Tth_rplC-rev2 (5′-GTGTGCTTCTGCG GCCAGATCTTCCGGC-3′) and sequenced using primers Tth_rplC-fwd1 and Tth_rplC_rev1. The *rrlA* and *rrlB* loci were amplified using primer pair Tth-23S-E (5′-CGCCAAGGAACTCTGCAAGTTGGC-3′) and Tth-23S-F (5′-CCAGAGGTGCGTCCCTTCCG GTCC-3′) and sequenced using primers Tth-23S-E and Tth-23S-F. Sequencing was performed at the University of Rhode Island Genomics and Sequencing Center.

To confirm that the mutations identified by sequencing were actually responsible for the resistance phenotypes, wild-type *T. thermophilus* was transformed with genomic DNA, selecting the original phenotype. Transformants were purified, gDNA was extracted, and the presence of the original mutations were confirmed by PCR and sequencing.

### 4.4. Disc Diffusion Assays

To measure antibiotic resistance, 100 μL of a saturated overnight culture (approximately 10^8^ cells) was plated onto a series of TMG plates. Onto the center of each plate was placed a 6 mm filter-paper disc (Whatman No. 2017-006) containing antibiotic. Concentrations used were as follows: tiamulin, erythromycin, clarithromycin, roxithromycin, azithromycin, midecamycin, tylosin, spiramycin, lincomycin, chloramphenicol, streptomycin, rifampicin, ciprofloxacin all 100 μg; and ampicillin, 20 μg. Plates were incubated overnight at 65 or 72 °C. The diameter of zones of inhibition were measured in millimeters.

## 5. Conclusions

The results described here provide a more comprehensive description of tiamulin-resistance mutations than previously observed in a single system. Given the amenability of ribosomes from *T. thermophilus* to crystallization and structure determination, these mutants should provide an important resource for establishing the precise mechanism of resistance to tiamulin at the atomic level.

## Figures and Tables

**Table 1:** Spontaneous tiamulin-resistant mutants identified in this study.

Strain	Genotype	Mutation	Tiamulin Selection	Temperature
IB-21	*rplC*-R149H	uL3-R149H	50 μg/mL	72 °C
IB-21	*rplC*-R149C	uL3-R149C	100 μg/mL	72 °C
HB27	*rplC*-R149C	uL3-R149C	100 μg/mL	72 °C
IB-21	*rrlAB*-G2061A	23S-G2061A	50, 100 μg/mL	72 °C
HB27	*rrlAB*-G2061A	23S-G2061A	100 μg/mL	65 °C
IB-21	*rrlAB*-G2061U	23S-G2061U	100 μg/mL	62 °C
HB27	*rrlAB*-G2061U	23S-G2061U	100 μg/mL	72 °C
HB27	*rrlAB*-A2451U	23S-A2451U	100 μg/mL	72 °C
IB-21	*rrlAB*-C2452U	23S-C2452U	50 μg/mL	72 °C
IB-21	*rrlAB*-U2500A	23S-U2500A	100, 200 μg/mL	72 °C
HB27	*rrlAB*-U2500A	23S-U2500A	50 μg/mL	65 °C
IB-21	*rrlAB*-U2504G	23S-U2504G	100 μg/mL	62 °C
HB27	*rrlAB*-U2504G	23S-U2504G	200 μg/mL	72 °C

*E. coli* numbering used throughout. Mutants were selected on TMG medium containing tiamulin at the indicated concentrations and at the temperature indicated.

**Table 2:** Cross-resistance phenotypes of tiamulin-resistant mutants.

Mutant	Zone of Inhibition (mm)													
	Tam	Ery	Clr	Rox	Azm	Mid	Tyl	Spi	Lin	Chl	Str	Rif	Amp	Cip
WT (IB-21)	30	27	36	32	22	21	36	16	36	29	14	17	40	22
WT (HB27)	32	29	36	33	25	24	37	15	41	33	16	-	38	21
uL3-R149H	16	23	33	28	18	25	39	10	44	38	15	17	45	20
uL3-R149C	17	25	36	32	22	23	38	12	35	31	17	16	51	25
23S-G2061A	-	42	63	65	34	46	71	40	34	-	20	27	55	28
23S-G2061U	-	22	35	24	11	24	48	13	7	9	17	17	50	25
23S-A2451U	-	12	33	28	15	28	64	27	-	-	30	21	50	26
23S-C2452U	-	14	25	20	10	20	48	13	20	9	16	15	42	22
23S-U2500A	-	14	20	19	11	12	36	7	18	15	15	17	40	21
23S-U2504G	-	18	34	28	13	18	46	16	22	-	16	16	42	22

All mutants are derived from IB-21 with the exception of the 23S rRNA-A2451U mutant, which is derived from HB27. Additionally, included are mutants obtained in a previous study, all derived from IB-21. Tam, tiamulin; Ery, erythromycin; Clr, clarithromycin; Rox, roxithromycin; Azm, azithromycin; Mid, midecamycin; Tyl, tylosin; Spi, spiramycin; Lin, lincomycin; Chl, chloramphenicol; Str, streptomycin; Rif, rifampicin; Amp, ampicillin; Cip, ciprofloxacin. Discs were 6 mm, such that a zone of 6 mm indicates no inhibition (indicated by -). Due to the high frequency of reversion, the G2061A mutant was grown in the presence of chloramphenicol. Blue, strong resistance (complete or nearly complete absence of inhibition zone); light blue, weak resistance (50% or greater reduction in inhibition zone).

**Table 3:** Closest approaches of 23S rRNA bases and tiamulin.

Mutation	Tiamulin Phenotype ^1^	Conservation	Nearest Neighbor	Distance (*Dra*, Å)	Distance (*Hma*, Å)
A2058G	S	98.33/42.28	A2058-N6:Tam-O2	7.1	7.1
A2059G	S	99.17/99.41	A2059-N1:Tam-O2	6.7	5.8
G2061A,U	R,R	100.00/99.88	G2061-N2:Tam-O4	3.0	3.0
G2447A	R	96.28/98.20	G2447-N1:Tam-C8	5.3	5.0
A2451U	R	100.00/100.00	A2451-N6:Tam-C7	3.6	3.3
C2452U	R	100.00/100.00	C2452-C1’:Tam-O1 C2452-O2:Tam-O1	3.9 4.0	4.7 2.9
A2453G	S	99.59/82.39	A2453-N3:Tam-C2	6.0	6.6
U2500A,C	R,S	100.00/99.64	U2500-O2:Tam-C1 U2500-O2:Tam-C2	7.2 7.4	6.4 6.1
A2503G	S	99.59/98.31	A2503-O2’:Tam-C17 A2503-C8:Tam-O2	3.0 -	4.0 3.0
U2504G,C,A	R,S,R	99.59/98.43	U2504-C5:Tam-C1 U2504-O2’:Tam-C2	3.5 5.0	5.6 3.0

^1^S, sensitive; R, resistant. Distance based on PDB entries 1xbp.cif for *D. radiodurans* (*Dra*) [8] and 3g4s.cif for *H. marismortui* (*Hma*) [10].
